# *Trypanosoma cruzi*-derived exovesicles contribute to parasite infection, tissue damage, and apoptotic cell death during *ex vivo* infection of human placental explants

**DOI:** 10.3389/fcimb.2024.1437339

**Published:** 2024-10-14

**Authors:** Alejandro Fernández-Moya, Bielca Oviedo, Ana Liempi, Jesús Guerrero-Muñoz, Cristian Rivas, Rocío Arregui, Sebastian Araneda, Alberto Cornet-Gomez, Juan Diego Maya, Marioly Müller, Antonio Osuna, Christian Castillo, Ulrike Kemmerling

**Affiliations:** ^1^ Instituto de Ciencias Biomédicas, Facultad de Medicina, Universidad de Chile, Santiago, Chile; ^2^ Instituto de Ciencias Naturales, Facultad de Medicina Veterinaria y Agronomía, Universidad de Las Américas, Santiago, Chile; ^3^ Departamento de Patología y Medicina Oral, Facultad de Odontología, Universidad de Chile, Santiago, Chile; ^4^ Facultad de Odontología y Ciencias de la Rehabilitación, Universidad San Sebastián, Santiago, Chile; ^5^ Departamento de Parasitología, Instituto de Biotecnología, Universidad de Granada, Granada, Spain; ^6^ Departamento de Tecnología Médica Facultad de Medicina, Universidad de Chile, Santiago, Chile

**Keywords:** *Trypanosoma cruzi*, placenta, tissue damage, infection, exovesicles

## Abstract

*Trypanosoma cruzi*, the causative agent of Chagas disease, can be congenitally transmitted by crossing the placental barrier. This study investigates the role of *T. cruzi*-derived exovesicles (TcEVs) in facilitating parasite infection and the consequent tissue damage and apoptotic cell death in human placental explants (HPEs). Our findings demonstrate that TcEVs significantly enhance the parasite load and induce tissue damage in HPEs, both in the presence and absence of the parasite. Through histopathological and immunohistochemical analyses, we show that TcEVs alone can disrupt the placental barrier, affecting the basal membrane and villous stroma. The induction of apoptotic cell death is evidenced by DNA fragmentation, caspase 8 and 3, and p18 fragment immunodetection. This damage is exacerbated when TcEVs are combined with *T. cruzi* infection. These findings suggest that TcEVs play a critical role in the pathogenesis of congenital Chagas disease by disrupting the placental barrier and facilitating parasite transmission to the fetus. This study provides new insights into the mechanisms of transplacental transmission of *T. cruzi* and highlights the potential of targeting TcEVs as a therapeutic strategy against congenital Chagas disease.

## 1 Introduction

Chagas Disease (CD), also known as American Trypanosomiasis, is caused by the protozoan parasite *Trypanosoma cruzi* (*T. cruzi*) and can be transmitted from mother to child through the placenta. Although approximately 60% of congenitally infected newborns are asymptomatic at birth, they exhibit a higher incidence of low Apgar scores, low birth weight, and prematurity than uninfected newborns. Some suffer from severe symptoms that can rapidly lead to death (Kemmerling et al., 2019; Carlier et al., 2020). Also, all congenitally infected infants are at risk of developing the disabling and life-threatening chronic phase of CD, highlighting congenital infection with *T. cruzi* as a significant global public health concern (WHO, 2015; Requena-Méndez et al., 2017; Buekens et al., 2018; Kemmerling et al., 2019; Carlier et al., 2020).

During congenital transmission, the parasite reaches the developing fetus by crossing the placental barrier (Duaso et al., 2010; Kemmerling et al., 2010; Liempi et al., 2016; Kemmerling et al., 2019; Carlier et al., 2020). The success of congenital transmission depends on complex host-pathogen interactions that include the placenta and the parasite (Liempi et al., 2016; Kemmerling et al., 2019; Carlier et al., 2020).

The placenta provides a metabolic exchange for the developing fetus and ensures normal embryo-fetal growth/development (Benirschke et al., 2012). Additionally, the placenta is a critical immunological mediator during pregnancy, shielding the fetus against several pathogens, including *T. cruzi* (Mor et al., 2017; Kemmerling et al., 2019; Carlier et al., 2020). Hence, the parasite must circumvent two main placental defense mechanisms at the free chorionic villi, the basic morpho-functional units surrounded by maternal blood in the intervillous space. The first line of defense is the trophoblast, a lining epithelium that covers the chorionic villi (Carlier et al., 2020). The second line of defense is the basal membrane, which separates the epithelium from the fetal connective tissue (villous stroma (VS)), which contains fibroblasts, macrophages, the fetal capillaries, and a net of extracellular matrix (ECM) (Duaso et al., 2010; Kemmerling et al., 2019; Carlier et al., 2020). The trophoblast, the basal membrane, and the VS constitute a physical barrier to pathogens in maternal blood. Nevertheless, in congenital CD (CCD), the parasite can overcome this barrier (McConkey et al., 2016; Carlier and Truyens, 2017; Heerema-McKenney, 2018; Kemmerling et al., 2019; Carlier et al., 2020).

*T. cruzi* possesses various virulence factors that allow it to reproduce and effectively evade host defenses and generate damage or morbidity in the affected individual (Jiménez et al., 2019). Parasite virulence is conferred by specific *T. cruzi* surface or secreted proteins that target host cells, facilitating parasite entry and replication. Interestingly, many *T. cruzi* virulence factors are secreted in extracellular vesicles and considered critical during host-pathogen interactions (de Pablos Torró et al., 2018; Torrecilhas et al., 2020; Rossi et al., 2021; Cornet-Gomez et al., 2023; Garcez et al., 2023). EVs modify the expression of certain genes involved in numerous cellular functions and act directly over the extracellular matrix (ECM) (de Pablos Torró et al., 2018; Torrecilhas et al., 2020; Cornet-Gomez et al., 2023; Garcez et al., 2023).

EVs are cell-derived membranous structures that encapsulate different biomolecules, including proteins, nucleic acids, lipids, and metabolites (Gill et al., 2019; Varikuti et al., 2020; Moreira et al., 2021; Rossi et al., 2021; Cornet-Gomez et al., 2023; Garcez et al., 2023), which play crucial roles in cell-to-cell interactions by transferring the encapsulated molecules and triggering diverse cellular responses upon uptake (de Pablos Torró et al., 2018; Torrecilhas et al., 2020; Moreira et al., 2021; Rossi et al., 2021; Cornet-Gomez et al., 2023; Garcez et al., 2023). In addition, cargo encapsulation within the EVs offers protection against extracellular enzymes and aqueous environments (Gill et al., 2019). EVs have also been recognized as significant mediators of transplacental infections (Kaminski V de et al., 2019). However, the potential role of *T.cruzi*-derived EVs (TcEVs) in placental infection has not yet been studied. In this study, we evaluated the role of exosomes derived from infective trypomastigote forms TcEVs during *ex vivo* infection of human placental explants (HPEs), analyzing their effect on the placental barrier and parasite DNA load. Our results reveal that the TcEVs increase parasite infection and independently induce tissue damage and apoptotic cell death in placental tissue, thereby contributing to the overall tissue damage caused by *T. cruzi*.

## 2 Materials and methods

### 2.1 Parasite culture and harvesting

Infective trypomastigote forms of *T. cruzi* were obtained by replicating part of their biological cycle in VERO cells (ATCC^®^ CCL-81). VERO cells were grown in RPMI medium supplemented with 5% fetal bovine serum (FBS) and antibiotics (penicillin-streptomycin). Semiconfluent VERO cells were incubated with a culture of Y-strain epimastigotes (a non-infective cellular form of the parasite) in the late stationary phase containing about 5% of infective trypomastigotes. Trypomastigotes invade fibroblasts and replicate intracellularly as amastigotes. After 72 hours, amastigotes transform into trypomastigotes, which lyse the host cells. The parasites were recovered by low-speed centrifugation (500 x g), producing trypomastigotes in the supernatant and amastigotes in the sediment (Villalta and Kierszenbaum, 1982).

### 2.2 EVs isolation and treatments

Briefly, *T. cruzi* purified trypomastigotes were incubated for 5 h at 37°C in RPMI medium (Sigma, USA) buffered with 25 mM HEPES at 7.2 and supplemented with 10% exosome-free IFBS. Afterward, parasites were removed by centrifugation at 3,500 x *g* for 15 min; the supernatant was collected and centrifuged at 17,000 x *g* for 30 min at 4°C to eliminate the apoptotic bodies and ectosomes and then filtered through a 0.22 μm pore filter (Sartorius, Germany) and ultracentrifuged at 100,000 x *g* for 16h to obtain the TcEVs. The resulting pellet was washed thrice in PBS by ultracentrifugation and resuspended in 100 μL PBS. To inactivate parasite virulence factors, TcEVs were incubated in a water bath at 80˚C for 30 min and then washed twice in PBS by ultracentrifugation at 100,000 x *g* for 1 hour (De Pablos et al., 2016; Retana Moreira et al., 2019). The size and concentration of *T. cruzi* EV samples were determined by measuring Brownian motion as a function of particle size using a NanoSight NS300 (Malvern Instruments, UK), a system equipped with an sCMOS camera and a 488 nm blue laser beam, as described previously (Cornet-Gomez et al., 2023). Inactivation of the TcEVs was assayed by protease enzymatic activity. Briefly, TcEVs were resuspended in a solution containing 1 ml of N-a-benzoyl-DL-arginine (BApNA) (0.2 mM) in 50 mM Tris-HCl (pH 7.4) and then 0.2 mM Dithiothreitol was added. The samples were incubated at 37°C for 30–60 min of reaction, and finally, the absorbance of each solution was measured at 405 nm (Soto et al., 2022). The iTcEVs did not show protease activity.

### 2.3 HPE culture and parasite infection

Human-term placentas were obtained from uncomplicated pregnancies from cesarean deliveries. Each patient gave informed consent for the experimental use of the placenta as stipulated by the Code of Ethics of the Faculty of Medicine of the University of Chile. The exclusion criteria for the patients were the following: major fetal abnormalities, placental tumor, intrauterine infection, obstetric pathology, or any other maternal disease. The organs were collected in a cold, sterile saline-buffered solution (PBS) and processed no more than 30 minutes after delivery. The maternal and fetal surfaces were discarded, and villous tissue was obtained from the central part of the cotyledons. The isolated chorionic villi were washed with PBS to remove blood, dissected into approximately 0.5 cm^3^ fragments (Duaso et al., 2010; Liempi et al., 2020), and co-cultured for 24 hours in the presence and absence of 0.2 ug/ml of TcEVs (equivalent to 0.284 x 10^8^ EVs/ml) or inactivated TcEVs (iTcEVs) and in the presence and absence of 10^5^ parasites/ml. In addition, HPE were also pre-incubated for 2 hours with TcEVs or iTcEVs and then challenged with *T. cruzi*.

### 2.4 DNA amplification by real-time PCR

Genomic DNA was extracted from the placental tissue with the Wizard Genomic DNA Purification Kit (Promega^®^) according to the manufacturer’s instructions and quantified by µDropPlate in a Varioskan Flash Multimode Reader (Thermo Scientific^®^). Two specific pairs of primers were used to amplify human and parasite DNA for hGADPH and *T. cruzi* satellite DNA (**Table 1**). Each reaction mix contained 0.5 µL at 10 nM of each primer (forward and reverse), 1 ng of DNA from samples, 10 µL of SensiFAST™ SYBR^®^ Hi-ROX Kit (Bioline^®^), and H_2_0 for a total of 20 µL. Amplification was performed in an ABI Prism 7300 sequence detector (Applied Biosystems^®^). The cycling programs were as follows: initial denaturation at 95°C for 3 min, followed by 40 cycles of 95°C for 5 s, 60°C for 30 s, and a dissociation stage was added, ranging from 60 to 95°C [29]. Relative quantification analysis of the results was expressed as RQ values by the comparative Control (ΔΔCt) method (Pfaffl, 2001; Castillo et al., 2012).

**Table 1:** Oligonucleotides are used as primers for genome amplification in qPCR analysis.

qPCR Primers	Primer Forward	Primer Reverse
***T. cruzi***	GCTCTTGCCCAC AMGGGTGC	CAAGCAGCGGAT AGTTCAGG
**hGAPDH**	TGATGCGTGTAC AAGCGTTTT	ACATGGTATTCACCA CCCCACTAT

### 2.5 Tissue sample processing and analysis

The HPE were fixed in 4% paraformaldehyde (PFA) in 0.1 M of phosphate buffer (pH 7.3) for 24 h, then dehydrated in alcohol, clarified in xylene, embedded in paraffin, and sectioned at 3 or 5 µm. Paraffin histological sections were stained with hematoxylin-eosin for routine histological analysis, trichrome and with picrosirius red–hematoxylin staining for collagen histochemistry, periodic acid-Schiff (PAS) and silver staining for carbohydrate-containing tissue elements [31]. In addition, standard immunoperoxidase techniques were used to show collagen IV (Cell Marque^®^ 239M-1, dilution 1:200 v/v), vimentin (Cell Marque^®^ 347M-1, dilution 1:200 v/v) and p18 fragment of active caspase 8 (Cell Signaling #9496S, dilution 1:200 v/v). Briefly, the primary antibodies were applied individually to each section for 1 h at 4°C (collagen IV), 1 h at 37°C (vimentin), or overnight (p18/caspase 8) respectively. Immunostaining was performed using a horseradish peroxidase-labeled streptavidin-biotin kit (ImmPACT™ DAB peroxidase substrate #SK-4105; Vector Laboratories) following the manufacturer’s directions using diaminobenzidine as the chromogen. Sections were counterstained with Mayer’s hematoxylin (ScyTek, Logan, UT, USA) and mounted with Entellan (Merck, Kenilworth, NJ, USA). Immunohistochemical controls were done by replacing the primary antibodies with phosphate-buffered saline (Duaso et al., 2010; Suvarna et al., 2018). All controls were negative. All sections were examined by light microscopy (Leitz Orthoplan, Leitz, Wetzlar, Germany) and images were captured with a Canon 1256 camera. The histopathological damage and basal membrane continuity were scored as described in **Table 2** (Gibson-Corley et al., 2013; Liempi et al., 2020). The chromogen intensity in immunohistochemistry and histochemistry was analyzed via reciprocal staining (Carrillo et al., 2016) or staining intensity (Duaso et al., 2011; Guerrero-Muñoz et al., 2023) using the Image J (http://fiji.sc/Fiji) software. For counting or histopathology, at least ten sections from different areas of three different slides in each experimental condition were used. We counted five squares of approximately 4,000 um^2^ of area in each section.

**Table 2:** Scores for histopathological and basal membrane analysis (PAS staining).

Score	Histopathology damage	Trophoblast basement membrane continuity score
1	Attached trophoblast, intact fetal connective tissue	Absent
2	Slight trophoblast detachment and/or fetal connective tissue disorganization	Almost absent
3	Almost complete detachment of trophoblast detachment and/or fetal connective tissue disorganization	Partially discontinuous
4	Complete detachment of the trophoblast and disorganization or destruction of the fetal connective tissue	Continuous

### 2.6 Caspase activity

Caspase-3 (Caspase-Glo^®^3/7 Assay, Promega^®^, Madison, WI, USA) and caspase-8 (Caspase-Glo^®^8 Assay, Promega^®^) enzyme activities were determined using commercial kits according to the manufacturer’s instructions. Briefly, Caspase-3 and 8 activities were determined through luminescence using luminogenic Caspase-3 (Ac-DEVD-pNA) or -8 (Ac-LETD-pNA) substrates, which reacts after cleavage by Caspase-3 or caspase-8 with a thermostable luciferase. Luminescence was read in a microplate reader (Varioskan^®^ Flash, Thermo Scientific). Data were normalized to the values obtained under control conditions (Carrillo et al., 2016). The intra-assay coefficients of variation were less than 10%.

### 2.7 DNA fragmentation using TUNEL assay

HPE samples were fixed in 4% (w/v) PFA in PBS pH 7.2 at 48°C for 24 h and then embedded in paraffin. Sections (5 µm) were made permeable with 20 mg/ml proteinase K for 10 min at room temperature, and the fragmented DNA was labeled using the TdT (terminal deoxynucleotidyl transferase) reaction mixture containing fluorescein-12-dUTP for 1 h at 37°C according to supplier recommendations (DeadEndTM Fluorometric TUNELsystem, Promega). Nuclei were stained with 1 mg/ml DAPI (4, 6-diamidino-2-phenylindole, Molecular Probes) and visualized in a Nikon Eclipse E400 epifluorescence microscope. Digital images of terminal deoxyuridine triphosphate nick end labeling (TUNEL) and nuclear morphology were obtained using a Digital DS-Ri1 Nikon camera (Nikon U.S.A., Melville, NY, USA). The apoptotic index was obtained by scoring TUNEL positive reaction in at least 500 nuclei (Duaso et al., 2011; Guerrero-Muñoz et al., 2023).

### 2.8 Statistics

All experiments were triplicated in at least 3 placentas. Results are expressed as means ± SD. The significance of differences was evaluated using Student’s t-test for paired data or by ANOVA followed by Dunnett’s post-test.

## 3 Results

### 3.1 TcEVs increase the parasite load and cause tissue damage in HPEs

**Figure 1:**
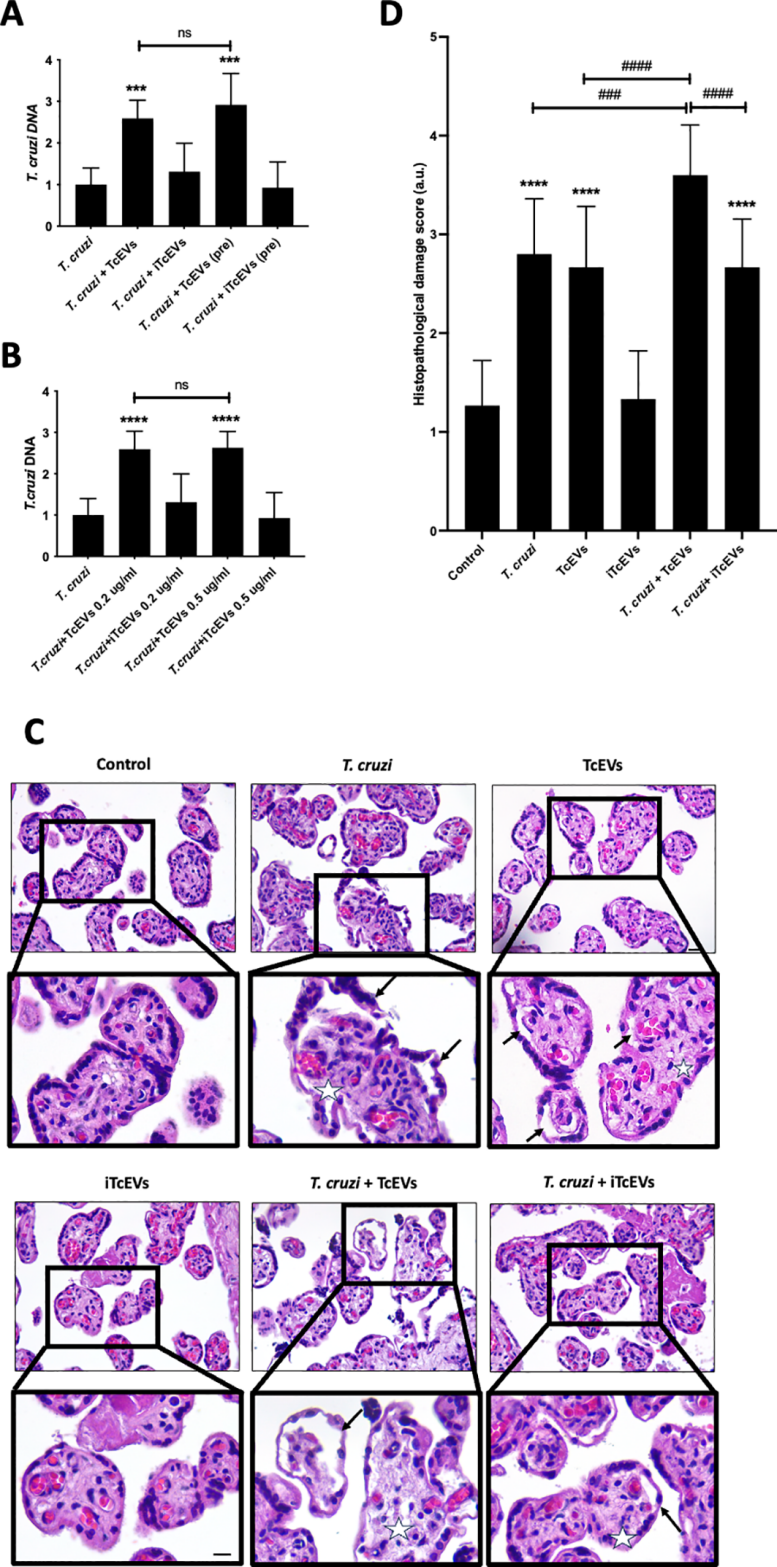
TcEVs increase the parasite load and cause tissue damage in HPEs. HPEs were co-cultured for 24 hours in the presence and absence of 0.2 ug/ml of TcEVs or iTcEVs and in presence and absence of 10^5^ parasites/ml. Additionally, HPE were also pre-incubated for 2 hours with TcEVs or iTcEVs and then challenged with *T. cruzi*. **(A)** In addition, HPEs were co-incubated with 0.2 ug/ml or 0.5 ug/mg of TcEVs **(B)**. The graphs present parasite DNA load data from real-time quantification using the ΔΔCt method. Co-incubated HPEs were further analyzed for histopathological damage **(C, D)**. The graph **(D)** quantifies the histopathological damage according to the parameters detailed in **Table 2**. All values are presented as mean ± S.D. from at least 3 independent experiments performed in triplicate. ***###p < 0.001; ****####p < 0.0001. ns: not significative. Bars marked with asterisks refer to conditions compared to control conditions; hashtags refer to comparisons between different experimental conditions.

HPEs were pre-incubated for 2 hours in the presence or absence of TcEVs or iTcEVs (0.2 ug/mL) and then co-cultivated with *T. cruzi* trypomastigotes for 24 hours (**Figure 1A**). Either the pre-incubation (291.88% ± 71.13, p ≤ 0.001) or co-incubation (259.28% ± 43.50, p ≤ 0.001) with TcEVs increase the parasite DNA load, showing no significant difference between both conditions (**Figure 1A**). We co-incubated the HPEs with 0.5 ug/mL of TcEVs or iTcEVs (**Figure 1B**). Either 0.2 ug/mL (259.28% ± 43.50, p ≤ 0.0001) and 0.5 ug/mL (262.73% ± 68.06, p ≤ 0.0001) of TcEVs concentrations increased the parasite DNA load with respect to the control condition, with no statistically significant differences between both experimental conditions. As expected, the inactivated TcEVs did not increase the parasite load in the placental samples (**Figures 1A, B**). Therefore, we performed the rest of the experiments by co-incubating the TcEVs with the HPEs. Histopathological analysis of the samples co-incubated with the parasite, using an arbitrary scoring system that considers parameters of trophoblast adherence and organization of the VS (**Table 2**; **Figures 1C, D**) was performed. **Figure 1D** shows that the control condition and the samples incubated with iTcEVs maintain the trophoblast adhered to the underlying ECM of the VS without or with minimal disruption of the epithelial continuity. In addition, the fibrillar organization of the ECM of the VS is also intact (**Figure 1D**), scoring 1.27 + 0.45 points (**Figure 1C**). However, in the presence of the parasite, destruction and detachment of the trophoblast (Arrows), as well as the disintegration of the ECM in the VS (Asterix), can be observed (**Figure 1D**) (2.80 + 0.56 points (p<0.0001), **Figure 1C**). Moreover, co-incubation of *T. cruzi* with TcEVs increases further the histopathological damage (3.60 + 0.50 points (p<0.0001) (**Figures 1C, D**)). Interestingly, detachment of the trophoblast is also visualized in the samples incubated only with TcEVs (2.66 + 0.61 points (p<0.0001)), suggesting that they can induce tissue damage by themselves.

### 3.2 TcEVs cause damage to the basal membrane that separates the trophoblast from the villous stroma in HPEs

**Figure 2:**
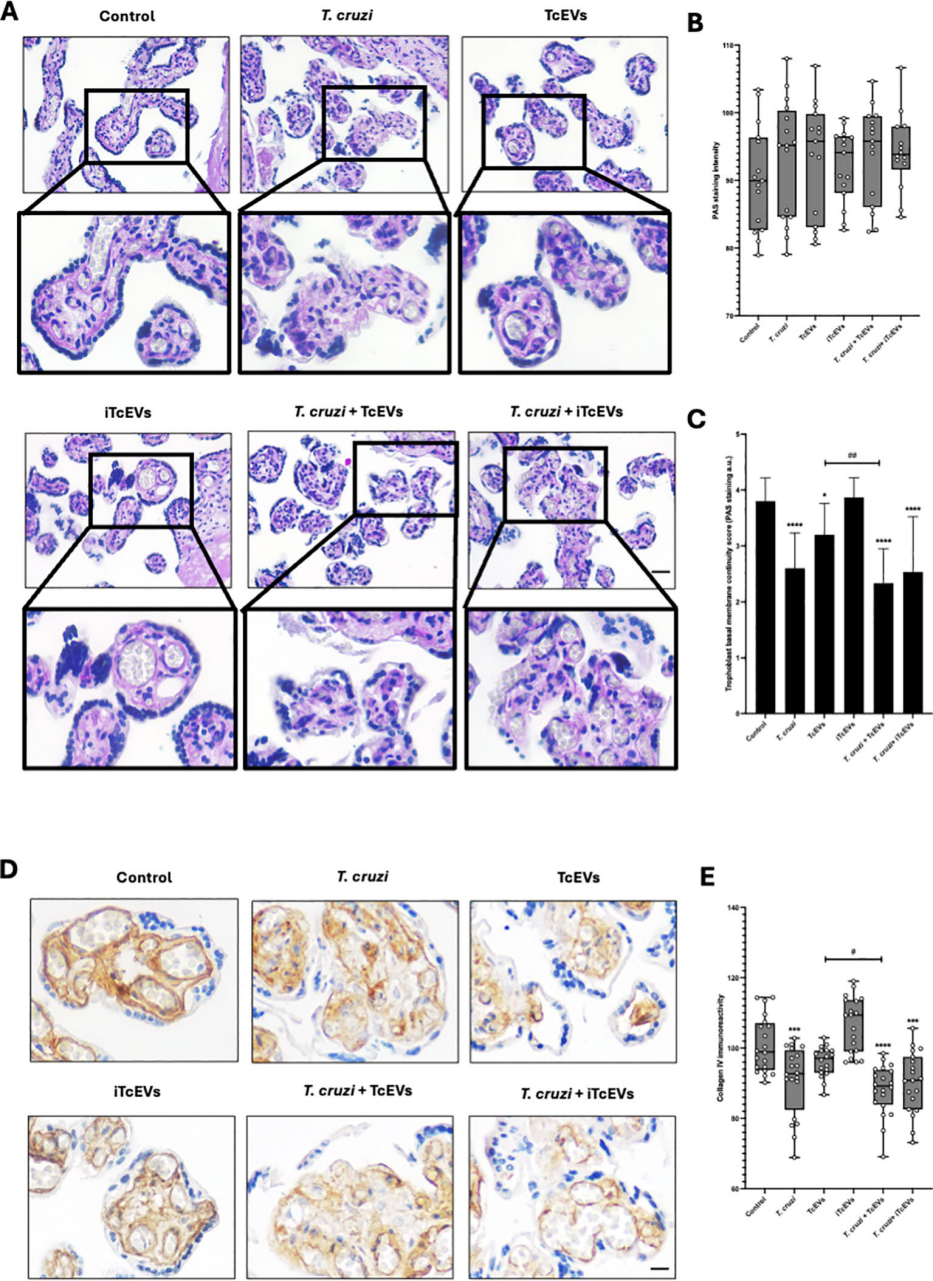
TcEVs cause damage to the basal membrane that separates the trophoblast from the villous stroma in HPEs HPEs were co-incubated for 24 hours in the presence or absence of TcEVs and iTcEVs, with or without *T. cruzi* trypomastigotes. Samples underwent routine histological analysis and were stained with PAS **(A)** and for collagen IV immunohistochemistry **(D)**. Graphs quantify PAS staining intensity **(B)**, scores basal membrane continuity **(C)** (**Table 2**), and collagen IV immunohistochemistry **(E)**. The data presented in panels **(B, D)** was analyzed using Image J software. All values are presented as mean ± S.D. from at least 3 independent experiments performed in triplicate. *#p < 0.05; ##p < 0.01; ***p < 0.001; ****p < 0.0001. Bars marked with asterisks refer to conditions compared to control conditions; hashtags refer to comparisons between different experimental conditions.

HPEs were incubated in the presence and absence of TcEVs, iTcEVs, or *T. cruzi* trypomastigotes for 24 hours. Periodic Acid-Schiff (PAS) histochemistry was analyzed using staining intensity and an arbitrary scoring system (**Table 2**). PAS staining is used to detect glycosylated molecules at the basal membranes and ECM of the VS (**Figures 2A–C**). We did not appreciate a significant difference regarding PAS staining intensity (**Figures 2A, B**). However, analyzing the basal membrane continuity, significant differences are evident. Thus, in the control samples (3.80 + 0.41 points) and those incubated with iTcEVs (3.87 + 0.35 points), a continuous magenta histochemical staining of the basal membrane that separates the trophoblast from the underlying VS, as well as those that separate the vascular endothelium of the fetal capillaries from the connective tissue, is appreciated. However, in the HPEs incubated in the presence of the parasite alone (2.60 + 0.63 points (p<0.0001), in the presence of *T. cruzi* and iTcEVs (2.53 ± 0.99 points (p<0.0001)), a noticeable loss of the continuity of the trophoblastic basal membrane was observed. In the case of co-incubation with the parasite and TcEVs, the increase of basal membrane discontinuity is also evident (2.3 ± 0.61 points (p<0.0001)). Similarly to the effect of the TcEVs described in the histopathological analysis (**Figures 2A, B**), TcEVs alone are also able to induce a mild but significant disruption of the continuity of the basal lamina (3.2 ± 0.56 points (p<0.05)), suggesting that TcEVs contribute to the basal laminae disorganization, facilitating tissue invasion.

Collagen IV is part of basal membranes and is present in the ECM of the placental chorionic villi (Duaso et al., 2010). Therefore, we analyzed the immunoreactivity for collagen IV (**Figures 2D, E**). Like the results for the basal membrane analysis by PAS staining, control samples (100.88 ± 7.95 points), and those incubated with iTcEVs (106.59 ± 7.85), a strong immunoreactivity is present. On the other hand, the incubation with TcEVs (96.99 ± 4.84) shows a slight but not significant decrease of the immunoreactivity. However, *T. cruzi* alone (90.96 ± 9.83 (p<0.001)) in the presence of iTcEVs (90.26 ± 8.76 (p<0.0001)) decreases significantly the detection of collagen IV which is more prominent in the presence of TcEVs ((88.12 ± 7.24 (p<0.0001 compared to the control sample, p<0.05 compared to the *T. cruzi* condition)).

### 3.3 TcEVs cause damage to the villous stroma present in HPEs, contributing to the *T. cruzi*-induced destruction of the placental barrier

**Figure 3:**
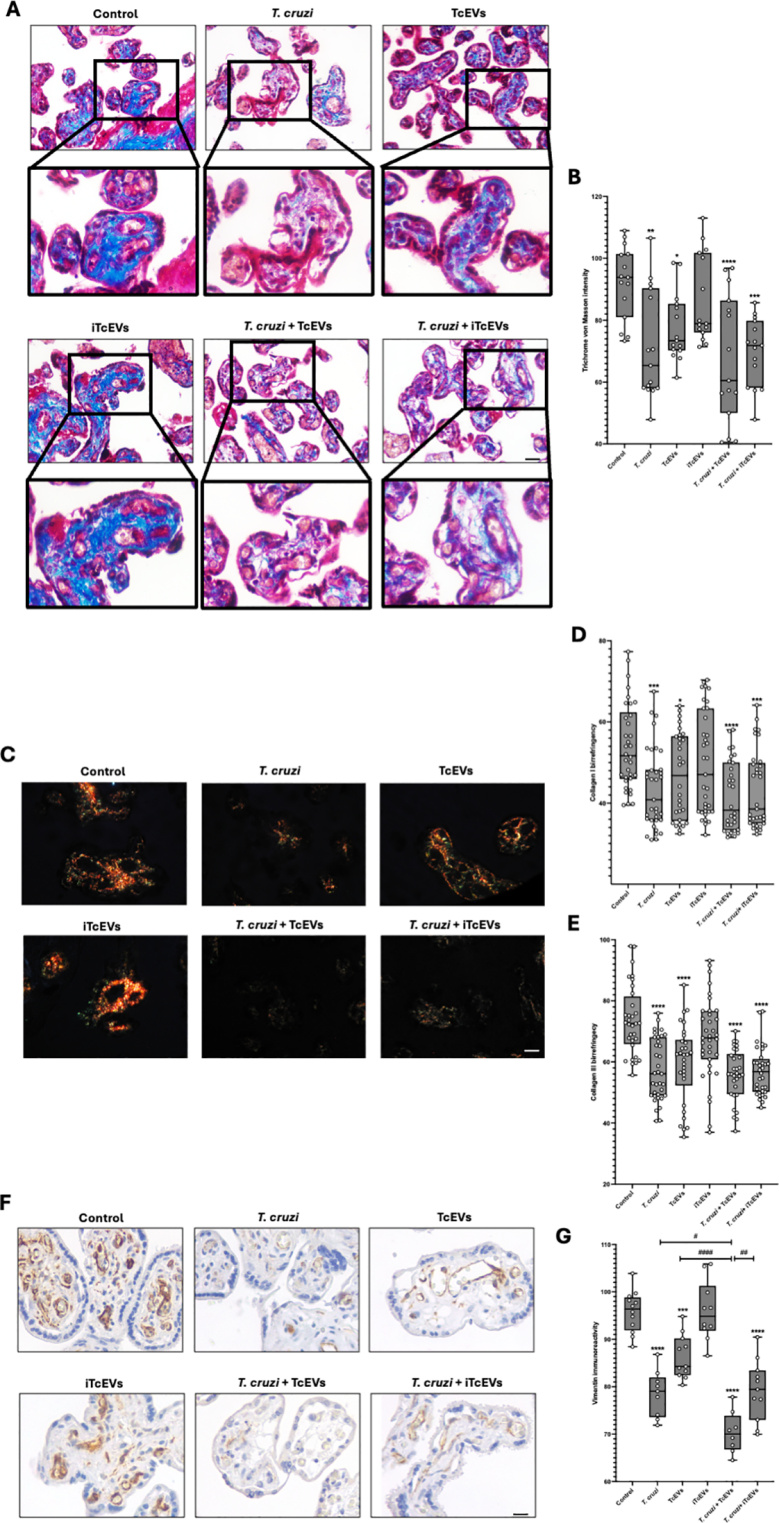
TcEVs damage the villous stroma in HPEs and contribute to the destruction of the placental barrier caused by *T. cruzi*. HPEs were co-incubated for 24 hours in the presence or absence of TcEVs and iTcEVs, with or without *T. cruzi* trypomastigotes. Samples underwent routine histological analysis and were stained with Masson’s Trichrome **(A)**, Picro-Sirius Red **(C)**, and vimentin immunohistochemistry **(F)**. Graphs show the quantification of collagen trichrome histochemistry **(B)**, collagen I **(D)**, and III **(E)** histochemistry, as well as vimentin immunohistochemistry **(G)** using the Image J software. All values are presented as mean ± S.D. from at least 3 independent experiments performed in triplicate. *#p < 0.05; **##p < 0.01; ***p < 0.001; ****####p < 0.0001. Bars marked with asterisks refer to conditions compared to control conditions; hashtags refer to comparisons between different experimental conditions.

We further performed histochemical analysis to visualize collagen fibers in connective tissues, processing the samples with Masson’s Trichrome stain (**Figures 3A, B**). Thus, control (92.24 ± 11.81) and HPE incubated with iTcEVs (86.67 ± 14.22) show intense blue staining, which decreases significantly in conditions incubated with TcEVs (77.62 ± 10.99 (p<0.05)), *T. cruzi* (71.78 ± 17.55 (p<0.01)), *T. cruzi* and iTcEVs (69.89 ± 10.85 (p<0.001)) or TcEVs (67.44 ± 20.63 (p<0.0001)).

Considering that the Masson’s Trichrome staining is unspecific, we performed the Picro-Sirius Red staining method to visualize collagen I and III (**Figures 3C–E**), which are observable, respectively, under polarized light with characteristic orange and green birefringence (**Figure 3C**). For collagen I (**Figure 3D**), the control condition (53.90 ± 10.53) and the one incubated with iTcEVs (49.69 ± 12-72) present a high orange birefringence under polarized light. However, in the experimental conditions incubated with the parasite alone (43.53 ± 9.92 (p<0.001)), in the presence of iTcEVs (43.34 ± 9.92 (p<0.001)) or TcEVs (41.74 ± 8.67 (p<0.0001)), a marked decrease in orange birefringence was observed, with barely distinguishable coloration under microscopy. As for the experimental condition incubated only with TcEVs (46.76 ± 10.61 (p<0.05)), a decrease in orange birefringence was also observed. The intensity of collagen III birefringences (**Figure 3E**) was 73.68 ± 11.35 and 67.73 ± 13.35 in control and HPEs incubated with iTcEVs, respectively. Samples incubated with either *T. cruzi* (57.29 ± 9.92), TcEVs (59.38 ± 12.81), the parasite in the presence of TcEVs (55.71 ± 8.45), or iTcEVs (56.90 ± 7.96) shows a significant (p<0.0001) decrease in collagen III birefringence. These results confirmed the high degree of disorganization of collagen types I and III, confirming the findings observed in Masson’s Trichrome staining (**Figures 3A, B**).

Vimentin is a marker of mesenchymal cells and is highly expressed in the intimate and media layer of fetal vessels and the perivascular area of chorionic villi (Sati et al., 2007). Since the last barrier that the parasite must overcome is the fetal vessels, we examined the immunoreactivity of vimentin in our samples (**Figures 3F, G**). Thus, a strong immunoreactivity in control (95.79 ± 4.37) and iTcEVs (95.93 ± 6.29) incubated samples can be observed. Contrarily, in samples incubated with the parasite alone (78.53 ± 4.87 (p<0.0001)), only with TcEVs (86.31 ± 4.60 (p<0.0001)) or in the presence of *T. cruzi* and iTcEVs (79.20 ± 6.38 (p<0.0001)), the immunoreactivity decreases significantly. Moreover, in samples incubated with the parasite and TcEVs (70.33 ± 4.36 (p<0.0001)), a further decrease of immunoreactivity for vimentin is observed.

### 3.4 TcEVs induce apoptotic cell death in HPEs

**Figure 4:**
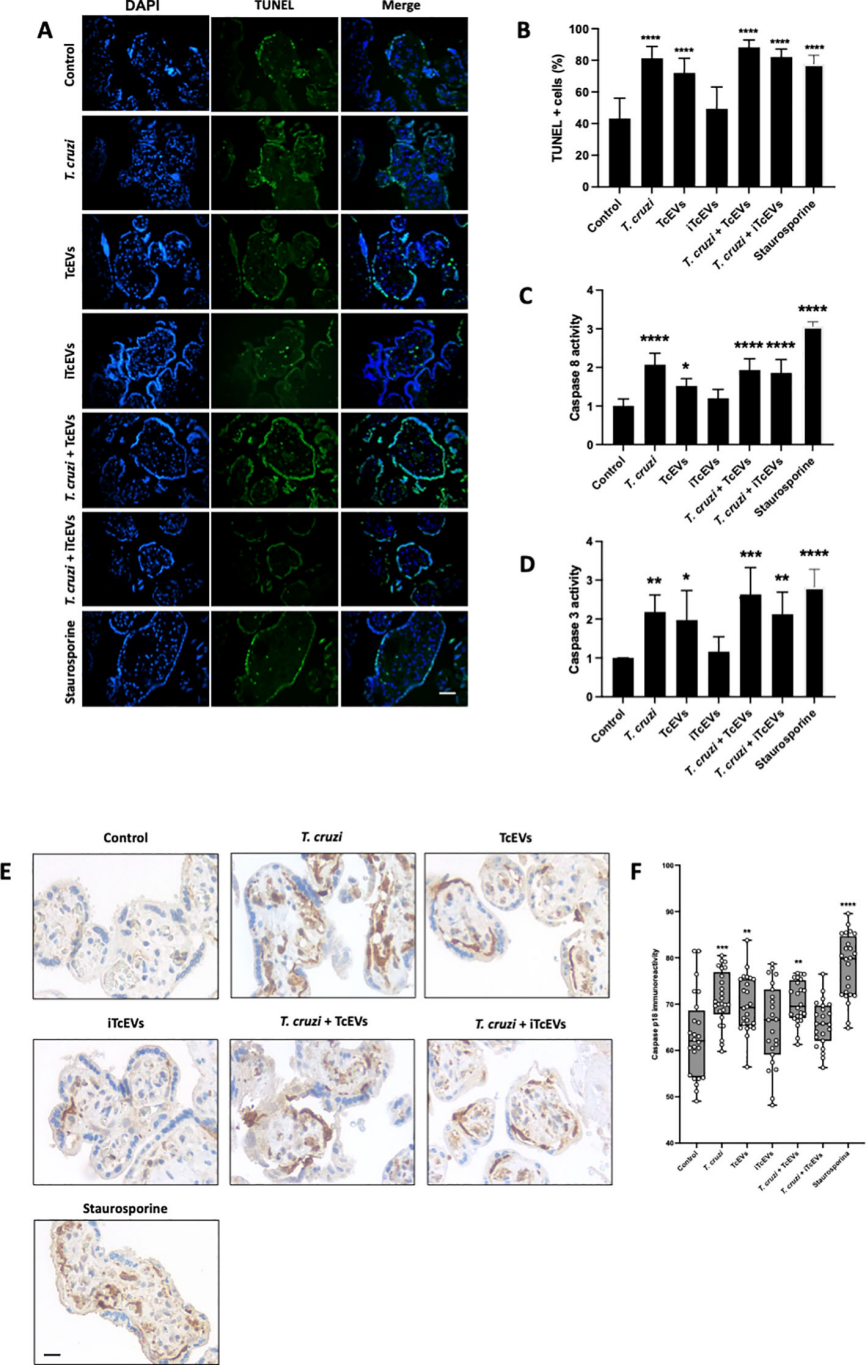
TcEVs induce apoptotic cell death in HPEs. HPEs were co-incubated for 24 hours in the presence or absence of TcEVs and iTcEVs, with or without *T. cruzi* trypomastigotes **(A)**. Staurosporine was used as a positive control for apoptotic cell death. DNA fragmentation was determined by the TUNEL method **(A, B)**. Graphs **(C, D)** display the percentage of TUNEL-positive cells. Caspase 8 **(C)** and caspase 3 **(D)** enzymatic activities were measured using commercial kits. The p18 active fragment of caspase 8 was detected by routine immunohistochemistry **(E, F)**, and the graph **(F)** quantifies the immunoreactivity and is analyzed with the Image J software. All values are presented as mean ± S.D. from at least 3 independent experiments performed in triplicate. *p < 0.05; **p < 0.01; ***p < 0.001; ****p < 0.0001.

*T. cruzi* induces apoptotic cell death in trophoblast cells (Carrillo et al., 2016) and HPEs (Duaso et al., 2011; Guerrero-Muñoz et al., 2023). Therefore, we evaluated the role of TcEVs in DNA fragmentation, caspase 8 activation and caspase 3 and 8 enzymatic activity (**Figure 4**). Thus, *T. cruzi* (81.20 ± 7.51% (p<0.0001)), TcEVs (70.07 ± 9.23% (p<0.0001)), *T.cruzi* in the presence of either TcEVs (88.20 ± 4.70% (p<0.0001)) or iTcEVs (82.10 ± 5.05% (p<0.0001)) increases significantly the percentage of TUNEL positive cells in a similar way than the positive control Staurosporine (77.82 ± 5.04% (p<0.0001)). Samples incubated with iTcEVs (49.37 ± 13.78%) do not show an increase of TUNEL-positive cells in comparison to the control samples (43.17 ± 12.95%) (**Figures 4A, B**).

We further evaluated the capacity of TcEVs to induce caspase enzymatic activity. The parasite (2.07 ± 0.29 (p<0.0001)) alone in the presence of TcEVs (1.93 ± 0.29 (p<0.0001)), iTcEVs (1.85 ± 0.34 (p<0.001)) can induce caspase 8 activity (**Figure 4C**). Moreover, TcEVs per se can induce caspase 8 activity (1.51 ± 0.19 (p<0.05)). As expected, iTcEVs (1.19 ± 0.23) do not induce caspase 8 activity. Regarding induction of the executioner caspase 3 activity, similar results were obtained (**Figure 4D**). Thus, *T. cruzi* in absence of TcEVs ((2.18 ± 0.43(p<0.01)) in presence of TcEVs (2.63 ± 0.69 (p<0.0001)) or iTcEVs (2.12 ± 0.57 (p<0.0001)) induce caspase 3 activity. In addition, TcEVs also induces the enzymatic activity (1.97 ± 0.75 (p<0.05)). Staurosporine, the positive control for apoptotic death induction, induces both caspase 8 (3.06 ± 0.11 (p<0.0001)) and 3 (2.82 ± 0.45 (p<0.0001)) activity. Finally, we analyzed the immunoreactivity of the p18 active fragment of caspase 8 as an apoptotic cell death marker. Thus, weak immunoreactivity in control (63.00 ± 9.08) and iTcEVs (65.93 ± 8.82) incubated samples can be observed. Contrarily, in samples incubated with Staurosporine (78.14 ± 6.90 (p<0.0001)), the parasite alone (71.14 ± 5.82 (p<0.001)) with TcEVs (70.36 ± 4.95 (p<0.0001)) or both (70.76 ± 4.26 (p<0.01)) the immunoreactivity increases significantly.

## 4 Discussion

The outcome of any infection is determined by the intricate interplay between host and pathogen (Lempke et al., 2023), and Chagas disease is no exception (Jiménez et al., 2019; Kemmerling et al., 2019). Congenital transmission of CD relies on several factors, particularly the placenta, the developing maternal and fetal immune systems and the parasite (Robbins and Bakardjiev, 2012; Kemmerling et al., 2019; Carlier et al., 2020). *T. cruzi* presents numerous virulence factors that facilitate adhesion, cellular and tissue invasion, immune response evasion, and replication and development within the host (Borges et al., 2016; Lantos et al., 2016; Prescilla-ledezma et al., 2022). Recent studies suggest that many parasitic virulence factors are released as cargo through EVs and are potentially more active than their soluble counterparts (Lantos et al., 2016; de Pablos Torró et al., 2018; Moreira et al., 2021; Garcez et al., 2023). Virulence factors present as TcEVs cargo from the infective trypomastigote form include the main *T. cruzi*-cysteine protease cruzipain (Cz) (Borges et al., 2016; Moreira et al., 2021), the GPI-anchored surface proteins transialidases (TSs) and mucin-associated surface proteins (MASPs) (Retana Moreira et al., 2019; Moreira et al., 2021), which are primarily localized on the surface of the EVs (Prescilla-ledezma et al., 2022).

The virulence factors in the TcEVs explain the increase of the parasite DNA load (**Figures 1A, B**) and the tissue damage observed during *ex vivo* infection of HPEs (**Figures 1C, D**, **2**, **3**). These results agree with the findings in other experimental models that have demonstrated increased cardiac parasitism, immunomodulation, and evasion of the host immune response (Trocoli Torrecilhas et al., 2009; Díaz Lozano et al., 2017; Garcez et al., 2023). Moreover, TcEVs enhance the infection of other protozoan organisms, such as *Toxoplasma gondii*, as evidenced by increased cells infected with tachyzoites (Retana Moreira et al., 2019). On the other hand, pathogens establish their presence in cells and tissues and induce molecular, cellular, and tissue changes (Duaso et al., 2010; Duaso et al., 2012; Lempke et al., 2023). In congenital transmission, *T. cruzi* must cross the placental barrier, initially encountering the trophoblast, which responds with various signaling mechanisms and cascades functionally interconnected (Duaso et al., 2010; Castillo et al., 2013; Carrillo et al., 2016; Liempi et al., 2019). For instance, in the presence of *T. cruzi*, the trophoblast modulates the ERK1/2 MAPK signaling pathway (Castillo et al., 2013), which can be activated by the surface glycoprotein gp85/TS present in the TcEVs (Retana Moreira et al., 2019; Moreira et al., 2021; Prescilla-ledezma et al., 2022). This glycoprotein can bind to intermediate filaments such as CK18 and vimentin with its FLY domain (Mattos et al., 2014) triggering a decrease in CK18 phosphorylation, thus redistributing these intermediate filaments in the affected cells (Tonelli et al., 2011), as well as inducing an increase in the ERK1/2 MAPK signaling cascade by significantly enhancing phosphorylation (Mattos et al., 2014). Moreover, the Cz, oligopeptidase B, and gp82/TS secreted by the parasite (Motta et al., 2012) and also present in Tryp-TcEVs trigger the mobilization of cytoplasmic Ca^2+^ and modulate the Rho GTPases, SUMO1 and 2 proteins leading to the destabilization of the actin cytoskeleton (Borges et al., 2016; Díaz-Luján et al., 2016; Cornet-Gomez et al., 2023), thereby improving *T. cruzi* invasion. Here, we also show that the parasite and the TcEVs decrease the immunoreactivity of vimentin, another important cytoskeleton component (**Figures 3F–G**).

TcEVs not only cause tissue damage *per se* in the HPE but also increase the parasite-induced one (**Figures 1C, D**). Therefore, our results show that TcEVs induce damage on their own in HPE, though milder compared to the parasite alone (**Figures 1**–**4**). These results are consistent with previous studies showing that *T. cruzi* induces destruction and detachment of the trophoblast depending on the parasitic load (Duaso et al., 2010; Díaz-Luján et al., 2016).

Importantly, the trophoblast is separated from the villous stroma by a basal membrane composed of different glycosylated, fibrous and anchoring proteins, including laminin, fibronectin and collagen IV (Duaso et al., 2010; Halfter et al., 2015; Carlier et al., 2020). The surface glycoprotein gp85/TS contains binding sites for fibronectin and laminin (Nde et al., 2006; Mattos et al., 2014). These proteins are subsequently degraded by virulence factors with peptidase activity, such as Cz, gp82/TS, and gp83/TS, allowing selective membrane degradation (Nde et al., 2006; Mattos et al., 2014). Here, we demonstrate that the parasite and TcEVs contribute to the degradation of the basal membrane (**Figures 2A, C**) particularly targeting its collagen IV component (**Figures 2D, E**). Other studies have shown that basal membrane components are critical during host-parasite interactions since silencing laminin expression inhibits parasite cell invasion (Nde et al., 2006).

The following component of the placental anatomical barrier is VS, where fetal capillaries are located. The VS is a connective tissue with an ECM composed mainly of collagen I and III (Duaso et al., 2010; Castillo et al., 2012; Duaso et al., 2012; Kemmerling et al., 2019; Carlier et al., 2020). We previously described that *T. cruzi* induces disorganization of type I collagen during *ex vivo* infection of HPE (Duaso et al., 2010), suggesting that the parasite reaches the fetal vessels, destroying the ECM through their proteases such as Cz (Santana et al., 1997; Duaso et al., 2010; Castillo et al., 2012) by inducing the host’s metalloproteinases, MMP-2 and MPP-9 (Castillo et al., 2012). Moreover, it has been shown that *T. cruzi* TS can modulate MMP-2 activation through the ERK1/2 signaling pathway (Musikant et al., 2021). Here, we show that TcEVs *per se* induce disorganization of the ECM, as evidenced by the less intense blue staining of Masson’s trichrome histochemical staining compared to the control (**Figures 3A, B**). This effect is also evident in the organization of collagen I and III, as seen by the birefringence reduction under polarized light microscopy (**Figures 3C–E**). These results might be related to the previously mentioned mechanisms, as the trophoblast’s detachment and the basal lamina’s discontinuity allow unimpeded access of the TcEVs to the VS, causing degradation and remodeling of the ECM.

Through the extrinsic pathway, *T. cruzi* induces apoptotic cell death in HPE (Robbins and Bakardjiev, 2012; Lempke et al., 2023) and the trophoblastic cell line BeWo (Sati et al., 2007). Interestingly, the caspase 8 pathway regulates the epithelial turnover of the trophoblast by regulating the differentiation of the basal cytotrophoblast cells (CTB) into the superficial syncytiotrophoblast (STB). This process triggers a cascade of events leading to the formation of syncytial apoptotic knots, which are subsequently released into the maternal blood (Gauster et al., 2009). Moreover, inhibition of caspases decreases the parasite load in BeWo cells (Carrillo et al., 2016). Here, we show that the TcEVs themselves induce DNA fragmentation (**Figures 4A, B**), caspase 8 activations (**Figures 4E–F**) and enzymatic activity (**Figure 4C**) as well as caspase 3 enzymatic activity (**Figure 4D**). Consequently, our results confirm previous studies demonstrating that the parasite induces apoptotic cell death to facilitate its invasion and suggest that the TcEVs play a fundamental role in this process. The *T. cruzi*-induced apoptotic cell death might be due to the presence of TS that mediates thymocyte depletion through the sialylation of endogenous acceptor molecules (Mucci et al., 2006). Moreover, since caspase 3 activity induces DNA fragmentation and cytoskeleton degradation (Yuan and Ofengeim, 2023), this likely explains the observed decrease in immunoreactivity of the intermediate filament vimentin following exposure to the parasite or the TcEVs. Interestingly, vimentin immunoreactivity diminishes in the endothelium of fetal capillaries or the surrounding cells (**Figure 3G**). Notably, vimentin has been shown to modulate the inflammatory response and apoptosis in *in vitro* models (Su et al., 2019).

## 5 Conclusion

We conclude that TcEVs contribute to *T. cruzi* tissue invasion during *ex vivo* infection of HPE and may play a role in the mechanism of the parasite’s congenital transmission. Furthermore, TcEVs represent potential targets for innovative therapeutic strategies against congenital Chagas disease.

## Data Availability

The raw data supporting the conclusions of this article will be made available by the authors, without undue reservation.
